# Farm data analysis for lifetime performance components of sows and their predictors in breeding herds

**DOI:** 10.1186/s40813-020-00163-1

**Published:** 2020-09-18

**Authors:** Yuzo Koketsu, Ryosuke Iida

**Affiliations:** School of Agriculture, Meiji University, Higashi-mita 1-1-1, Tama-ku, Kawasaki, Kanagawa 214-8571 Japan

**Keywords:** Farm data, Fertility, Lifetime efficiency, Lifetime performance, Longevity, Prolificacy

## Abstract

Our objectives in this review are 1) to define the four components of sow lifetime performance, 2) to organize the four components and other key measures in a lifetime performance tree, and 3) to compile information about sow and herd-level predictors for sow lifetime performance that can help producers or veterinarians improve their decision making. First, we defined the four components of sow lifetime performance: lifetime efficiency, sow longevity, fertility and prolificacy. We propose that lifetime efficiency should be measured as annualized piglets weaned or annualized piglets born alive which is an integrated measure for sow lifetime performance, whereas longevity should be measured as sow life days and herd-life days which are the number of days from birth to removal and the number of days from date of first-mating to removal, respectively. We also propose that fertility should be measured as lifetime non-productive days, whereas prolificacy should be measured as lifetime pigs born alive. Second, we propose two lifetime performance trees for annualized piglets weaned and annualized piglets born alive, respectively, and show inter-relationships between the four components of the lifetime performance in these trees. Third, we describe sow and herd-level predictors for high lifetime performance of sows. An example of a sow-level predictor is that gilts with lower age at first-mating are associated with higher lifetime performance in all four components. Other examples are that no re-service in parity 0 and shorter weaning-to-first-mating interval in parity 1 are associated with higher fertility, whereas more piglets born in parity 1 is associated with higher prolificacy. It appears that fertility and prolificacy are independent each other. Furthermore, sows with high prolificacy and high fertility are more likely to have high longevity and high efficiency. Also, an increased number of stillborn piglets indicates that sows have farrowing difficulty or a herd health problem. Regarding herd-level predictors, large herd size is associated with higher efficiency. Also, herd-level predictors can interact with sow level predictors for sow lifetime performance. For example, sow longevity decreases more in large herds than small-to-mid herds, whereas gilt age at first-mating increases. So, it appears that herd size alters the impact of delayed gilt age at first-mating on sow longevity. Increased knowledge of these four components of sow lifetime performance and their predictors should help producers and veterinarians maximize a sow’s potential and optimize her lifetime productivity in breeding herds.

## Background

Farm data are collected and stored on a daily basis by recording software in breeding herds. However, most producers only use the data to generate basic reports such as sow cards, working lists and a brief performance summary, and so do not use their farm data to its full potential [1]. Herd management based on farm data analysis [1] can help producers and veterinarians to maximize lifetime reproductive potential of sows to improve economic inefficiency [2–4]. Also, farm data analysis can accurately monitor lifetime performance in individual sows including the four components of longevity, prolificacy, fertility and lifetime efficiency measures, which were well used, but were not well organized to understand.

As a simple benchmark, the number of piglets weaned per sow per year (PSY) has been commonly used for monitoring changes in performance within a herd or for comparing PSY between herds [4, 5]. However, even though PSY is a good measurement for herd productivity in the short term, it is not the best measurement for sow lifetime performance including longevity [2, 4, 5]. Also, sow lifetime performance and inter-relationships between the four components have not been well studied, even though PSY and inter-relationships between key components are well known as a productivity tree [4, 5].

Also, sow lifetime performance can be predicted to a certain degree using reproductive performance factors [5] which are sow level predictors and herd-level predictors. Sow and herd-level predictors include age at first-mating, re-service in parities 0 and 1, the number of pigs born alive at parity 1, the number of stillborn piglets at parity 1, weaning-to-first-mating interval in parity 1, culling guidelines, timing of insemination and high-performing herds [4, 5]. Using such predictors, producers and veterinarians could maximize a sow’s potential and optimize her lifetime productivity. Therefore, this review aims to characterize the four components of sow lifetime performance, to propose inter-relationships between the four components and other key measures, and to summarize predictors for high lifetime performance of sows. Also, statistical methods for farm data analysis at herd-level or sow level have been developed over the last four decades using observational study concepts in epidemiology [5]. However, farm data analysis, especially for individual sow data, is still not well recognized as a valid scientific approach. Therefore, in this review we will emphasize the potential benefits of these methods.

## Review

### Limitation of piglets weaned per sow per year (PSY) as benchmark

PSY has been used widely as an integrated herd productivity measurement in North America for three decades [4, 6]. In the calculation, PSY is simply defined as the number of pigs weaned per litter multiplied by the number of litters farrowed per sow per year. However, it does not include any measure of longevity. In fact, more PSY is not directly associated with higher longevity, measured as the mean parity of removed sows or mean sow life days [7]. Also, some producers tend to cull low parity sows with fewer PBA to increase their herd’s PSY [8]. However, this means that the sows culled at low parity will not be able to realize their latent reproductive potential during herd-life. Also, high sow longevity can increase the profit per sow because lifetime PW by parity 3 is sufficient to recover the initial cost of a replacement gilt [2].

### Components of sow lifetime performance in breeding herds

Sow lifetime performance can be categorized as the four components of longevity, prolificacy, fertility and lifetime efficiency of sows. Longevity is measured as the number of parity at removal [2–4], herd-life days [9, 10] or sow life days. Sow life days is the number of days from birth to removal, whereas herd-life days is the number of days from the date that a sow is first-mated as a gilt to the removal date. In order to compare herd-life days between herds, it should begin from the day that gilts are first-mated, because the age of gilt entry to a herd can vary from the first day of a gilt piglet is weaned to the day that the replacement gilt is first-mated. Also, removal can be due to culling, death, euthanasia or being transferred.

A sow’s prolificacy should be measured as lifetime piglets born alive from first farrowing to removal. Fertility can be represented as herd-life nonproductive days, which contains the weaning-to-first-mating interval during herd-life, plus the re-service interval from the date that a sow is first-mated as a gilt until its removal [4, 5].

Sow life or herd-life annualized piglets born alive (PBA) is a measurement of sows’ lifetime efficiency that includes prolificacy, fertility and longevity [5]. Another measurement of sows’ efficiency is sow life or herd-life annualized piglets weaned (PW), which combines annualized PBA with lactational performance [5]. Annualized PBA simply combines prolificacy, fertility and longevity, whereas annualized PW contains additional lactational performance factors such as pre-weaning mortality, sow milk yields, nursing behavior and management practices (e.g. nursing and fostering techniques), that will reflect management and facility impacts.

**Fig. 1:**
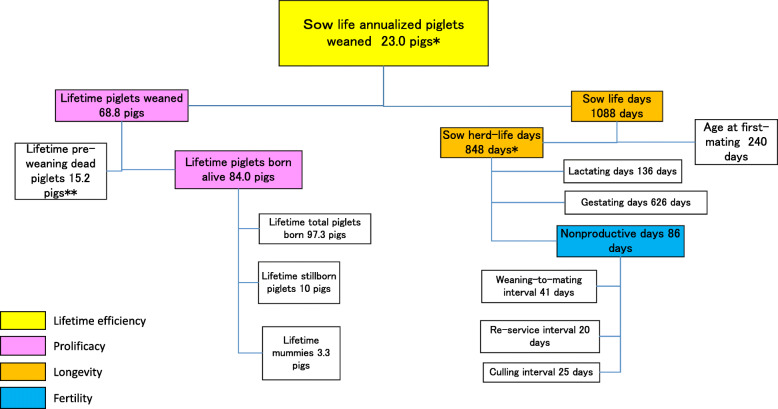
A lifetime performance tree for sow life annualized piglets weaned of 23.0 pigs. *Herd-life annualized piglets weaned is 29.6 pigs. **Average pre-weaning mortality is 18.1%

**Fig. 2:**
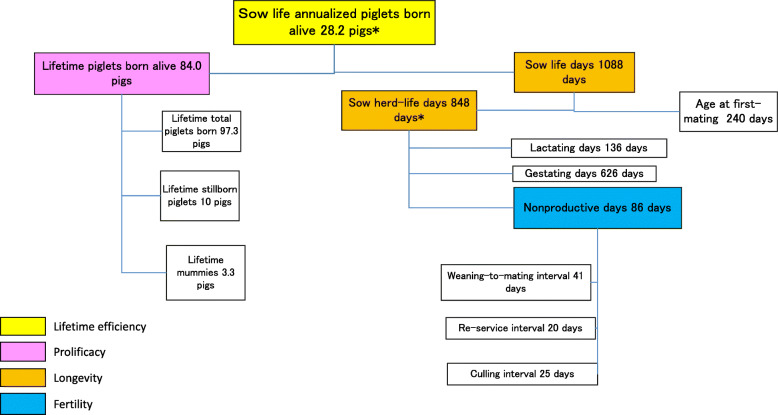
A lifetime performance tree for sow life annualized piglets born alive of 28.2 pigs. *Herd-life annualized piglets born alive is 36.2 pigs

Figure 1 shows the inter-relationships between the four components and other key measures for sow life annualized PW using theoretical value examples. Sow life annualized PW is the product of two components: sow life days and lifetime PW. Sow life days is the number of days from birth to removal, which is also derived from age at first-mating (AFM) plus herd-life days. Herd-life days includes lactating days, gestating days and nonproductive days. Herd-life annualized PW is also obtained from herd-life days and lifetime PW. In addition, the number of farrowings or the number of parity at removal can be roughly estimated by dividing gestating days during herd-life by 115 days (e.g. 626 gestating days divided by 115 is 5.4). Additionally, Fig. 2 shows the inter-relationships between the four components and other key measures for sow life annualized PBA. Sow life or herd-life annualized PBA is produced from lifetime PBA and either sow life days or herd-life days.

#### Sow longevity

Longevity of sows is commonly measured as the number of parity at removal [2–5]. In North America, Japan, Sweden and Spain, the mean parity at removal varies between 3.3 and 5.6 [11–15]. However, parity at removal is not an accurate way to monitor sow longevity because the parity at removal does not take the number of sow days into account, which can vary between herds for sows in the same parity. Therefore, sow life days or herd-life days should be used to measure longevity.

Mean herd-life days varies between 467 and 969 days in the U.S.A., the E.U. and Japan [9, 11, 16]. Longevity can be also measured as sow life days. Studies have shown mean sow life days ranging between 992 and 1088 days in the E.U. and Japan [10, 11].

#### Prolificacy

Prolificacy is represented by lifetime PBA, from the first farrowing to removal. Also, prolificacy depends on the number of parity at removal because the number of PBA increases with each farrowing up to parity 5 [13, 17]. Prolificacy is affected by genetics and breeding management including timing of insemination, semen quality and the stockman’s skill [4, 18]. Also, prolificacy per litter, measured as the number of PBA per litter, is limited by ovulation rate and embryonic survival [19], but there has been significant improvement in prolificacy over the last few decades due to genetic progress in the swine industry [3, 20]. Prolificacy can also be decreased by abortions and deaths, because these reduce sow longevity.

#### Fertility

Fertility is commonly measured as the number of litters farrowed per sow during a certain period such as a year [4]. However, over 50% of sows live for several years in a herd, so using the number of litters per sow over a period of 1 year is probably not the best way to accurately measure lifetime fertility because it will not take account of all the factors that could affect a sow’s fertility throughout its herd-life. Sow fertility can also be measured by lifetime nonproductive days which is directly associated with the number of litters farrowed in sow herd-life [4]. Sow lifetime nonproductive days is derived from re-service interval, weaning-to-first-mating interval and culling interval [5, 7]. Re-service interval and weaning-to-first-mating interval account for approximately 60% of the nonproductive days [21]. In particular, weaning-to-first-mating interval is closely related to the key reproductive hormone LH which mainly controls the fertility of sows [22, 23]. Also, re-service interval is related to occurrences of conception or pregnancy failure. Abortions in gilts and sows will also increase nonproductive days such as the re-service interval or culling interval [24]. Another factor critical for fertility is sow mortality, because increased mortality increases death interval, i.e. the number of days from a last event to the pig’s death, such as last-weaning-to-death interval of sows or last-mating-to-death interval of gilts, which increases nonproductive days.

#### Lifetime efficiency based on piglets weaned (PW) or piglets born live (PBA)

Annualized PW is a measure of sow lifetime efficiency calculated from lifetime PW divided by sow life days or herd-life days × 365.25 (Fig. 1). Also, annualized PBA is a measure of sow efficiency calculated from lifetime PBA divided by sow life days or herd-life days × 365.25 (Fig. 2).

It should be noted that the herd-life annualized PW of a sow can be very high if the sow is culled immediately after weaning in parity 1. For example, culling a parity 1 sow immediately after weaning 14 piglets at 22 days of age would produce a herd-life annualized PW of 37: 14 PW /(115 + 22) days × 365.25 days. This is a major drawback of using herd-life annualized PW. Culling a sow in parity 1 does not decrease the herd-life efficiency of the culled sow, but it does decrease sow efficiency measured as sow life annualized PW. For example, if the parity 1 sow mentioned earlier in this paragraph had AFM 240 days, then culling the sow in parity 1 would produce only 13.6 sow life annualized PW: 14 PW/(240 + 115 + 22) days × 365.25 days. It would also only produce sow life annualized PBA of 13.6 pigs, if this parity 1 sow, that had farrowed PBA 14 pigs, was culled immediately after weaning.

### Sow level predictors for lifetime performance

There are some measures of reproductive performance which are also predictors for sow lifetime performance [5]. Sow lifetime reproductive performance can be predicted to a certain degree using these performance factors.

#### Age of gilts at first-mating (AFM)

In North America, Spain, Portugal, Italy and Japan, the typical AFM is approximately 240 days, ranging between 160 and 370 days [4, 5, 25]. It is currently recommended to start mating gilts from 203 days of age in order to let replacement gilts gain enough body weight and body fat for first-mating [26].

Ages of gilts at first estrus can be recorded in major software with dates of heat-no-service events. The age of gilts at first estrus indicate age of puberty with the first ovulation, and early matured gilts with early age at first estrus have been associated with high lifetime performance [3, 27]. So, age of gilts at first estrus and dates of heat-no-service events are useful for predicting sow lifetime performance. However, neither measurement is commonly recorded in commercial herds [3, 27], whereas AFM is well recorded. Therefore, AFM is an important factor in farm data analysis for predicting lifetime performance of sows in breeding herds.

Late AFM has been associated with decreased longevity (i.e. removed at lower parity or shorter herd-life days), decreased prolificacy (i.e. fewer lifetime PBA), decreased fertility (i.e. more nonproductive days during herd-life) and decreased efficiency (i.e. fewer annualized PW or PBA) [11, 25]. For example, a study showed that sows first mated at 278 days of age or later were removed at lower parity and had fewer lifetime PBA than those mated at 229 days or earlier of age [27]. Late AFM has also been associated with more occurrences of late returns to service [28], which could be because late maturing gilts have poorly developed ovary and corpora lutea functions, and low progesterone concentrations [29]. Also, studies have shown that late AFM sows had a longer weaning-to-first-mating interval than early AFM sows, and so consequently prolonged nonproductive days or low fertility [25, 30]. Conversely, other studies showed that late AFM sows produced a small increase of 0.3–0.4 PBA when AFM increased by 100 days [31, 32].

Late AFM sows are also likely to become low-efficiency sows as a result of re-service interval or culling interval. Additionally, low lifetime performance of late AFM gilts is related to being overweight at breeding [31] which negatively affects sow longevity and lifetime efficiency such as annualized PW or PBA.

#### Body weights of gilts

The reproductive system of gilts is not fully matured, so appropriate nutrition management is needed during gilt development to provide them with the nutrients and energy necessary to achieve the target growth rate and body reserves [26, 33, 34]. A body mass of > 180 kg after farrowing is recommended to protect against the detrimental effects of fat and lean tissue loss during the first lactation on subsequent reproductive performance [35, 36]. Thus, if gilts are mated when they are at 135 to150 kg, they would be at the target weight at farrowing assuming a weight gain of 35 to 40 kg during gestation [3].

#### Re-service in parities 0 and 1

Return-to-service is a common occurrence in breeding herds. Approximately 10% of first-serviced sows do not conceive or conceive but fail to maintain pregnancy, and are then re-serviced. Furthermore, their farrowing rates decrease by 10% with each re-service [37]. The same study also found that re-serviced gilts had greater herd-life nonproductive days (124 vs. 175 days), lower parity at culling (4.2 vs. 3.8) and fewer herd-life PBA (46.2 vs. 44.2 pigs) than non-return gilts [37]. Furthermore, other studies have also found negative effects of return-to-service in gilts. For example, 41% of first-returned gilts had a second return in later life, compared with only 9% of returned sows in parity 6 having a second return [28]. Also, 36% of re-serviced parity 1 sows had one or more returns in later life [28], and re-serviced parity 1 sows had lower farrowing rates in subsequent parties than non-return sows [37–39].

Returned sows tend to have short estrus duration or weak estrus signs [40], which make it hard to detect estrus signs for appropriate timing of inseminations [18, 37, 41]. Also, any return occurrence increases a sow’s nonproductive days and decreases its fertility and efficiency. Therefore, returned sows should be closely monitored, because re-servicing the returned sows is an opportunity to improve sow efficiency [39, 41]. A general rule is to cull gilts or sows having two repeats, and in addition we recommend providing careful day-to-day management to monitor mated gilts and sows at risk of having a return [4, 41].

#### Number of piglets born alive (PBA) in parity 1

The number of PBA in parity 1 is an early predictor of sow prolificacy. In a study, sows were categorized into 4 groups based on the 10th, 50th and 90th percentiles of PBA in parity 1, and the sow group that had the most PBA in parity 1 produced more annualized PBA than the group that had fewest PBA in parity 1 [25, 42]. A sow’s prolificacy can be predicted by its PBA in parity 1, suggesting that prolificacy could be increased by gilt development and breeding programs and genetic potential [3, 43]. So, a gilt development unit is important to raise more highly prolific sows. However, E.U. and Japanese studies have not found any difference in weaning-to-first-mating interval or 21-day adjusted litter weights between the prolificacy groups based on PBA in parity 1 [25, 42].

#### Birth weight, weaning weight and pre-weaning growth rate

Highly prolific sows in genetic parent stocks can be a concern with regard to future replacement gilts. This is because more PBA in a litter is associated with a lower birth weight, and replacement gilts with a low birth weight will have compromised growth, reproductive performance and sow longevity [3, 44, 45]. Birth weight and pre-weaning growth rate in piglets to be replacement gilts are characteristics of litter-of-origin for lifetime performance of sows [44]. Lower pre-weaning growth rate is related to lower post-weaning growth performance [45], late puberty [44] and consequently late AFM.

Gilts with a low birth weight are associated with intrauterine growth retardation [46], and have fewer medium size follicles and more atretic follicles on the ovary than gilts with a high birth weight, when approaching expected puberty age [47]. Therefore, gilts with a low birth weight should not be selected, nor should those born to sows that have farrowed a large litter with large variation in birth weights. In addition to reproductive performance, high weaning weight and high pre-weaning growth of gilt piglets are associated with high survival and good performance as replacement gilts [48].

#### Use of nurse sows

While the use of foster-in and nurse-sow techniques can increase the number of PW and produce heavier litter weights at weaning, it could decrease the foster or nurse sow’s post weaning reproductive performance due to increased loss of body reserves and impaired metabolic state in the lactating sow [22, 49]. However, it has been found that nurse sows had farrowing rates and PBA similar to non-nurse sows in any parity, and the only difference was that they had a prolonged weaning-to-first-mating interval [50]. Consequently, the nurse sows produced 3–7 more herd-life annualized PW than non-nurse sows. However, it should be noted that the nurse sows appear to have been chosen as suitable sows to nurse another litter based on having good body reserves, nursing behavior and high feed intake during lactation.

#### Number of stillborn piglets in parity 1

Stillborn piglets are theoretically defined as piglets that are alive at the initiation of farrowing but die intrapartum [4], whereas, in practice, any piglet found dead behind a sow at the first check up after parturition, with no sign of mummification, is categorized as a stillborn piglet [51].

The number of stillborn piglets in parity 1 can be related to other aspects of sow lifetime performance. An increased number of stillborn piglets has been associated with decreased 21-day adjusted litter weights [52], more occurrences of uterine prolapse [53] or abortions at subsequent pregnancy [24], decreased farrowing rate, decreased PBA at subsequent parity, low prolificacy, low milk yields and probably low longevity due to low fertility [52]. Such negative associations of stillborn piglets are related to difficult farrowing, large litter size and shorter gestation length as sow factors and herd health conditions as environment factors [51, 54]. Therefore, supervision and assisted farrowing can help sows with difficult farrowing to overcome uterine inertia [51, 55]. In addition, caring for piglets by drying and warming them, and clearing their airways can help to reduce the number of stillborn piglets [54, 55]. Also, it is advisable to have herd health programs, especially for parity 1 sows, to reduce the risk of having stillborn piglets due to such infectious diseases. Antibiotic treatments are recommended for sows that have farrowed many stillborn piglets or that had dystocia [54, 56]. Moreover, it is important to note that the repeatability of sows’ farrowing stillborn piglets is low, and so there is no justification for culling low-parity sows based on the number of stillborn piglets in parity 1 [52].

#### Weaning-to-first-mating interval in parity 1

**Fig. 3:**
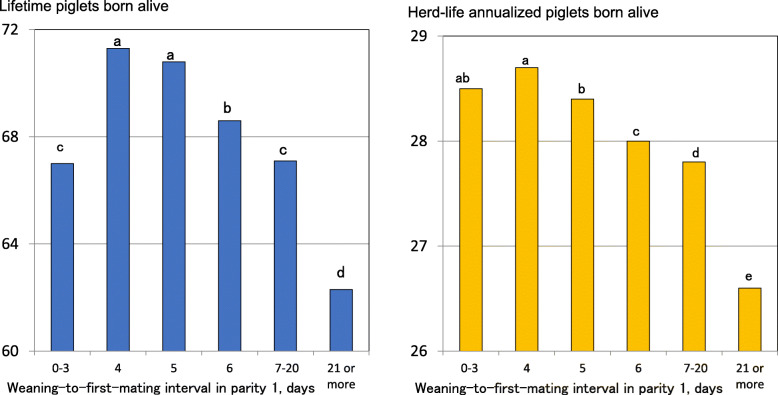
Comparisons of lifetime performance of sows between six weaning-to-first-mating interval groups in parity 1 [58]. ^a–e^Different superscripts represent significant differences in means

Parity 1 sows with weaning-to-first-mating interval 7–20 days have lower farrowing rates and fewer PBA in subsequent parities than those with weaning-to-first-mating interval 3–6 days [38, 57]. Another study showed that parity 1 sows with weaning-to-first-mating interval 4 days had 0.3 more herd-life annualized PBA than those with weaning-to-first-mating interval 5 days (Fig. 3). So, it suggested that parity 1 sows with a weaning-to-first-mating interval 4 days were the most efficient [58]. Also, the same study showed that there was little difference in herd-life annualized PBA between parity 1 sows with a weaning-to-first-mating interval 0–3 days and those with weaning-to-first-mating interval 4 and 5 days. So, it suggests that parity 1 sows with weaning-to-first-mating interval 0–3 days were capable of having the same lifetime efficiency as those with weaning-to-first-mating interval 4 or 5 days.

Prolonged weaning-to-first-mating interval is related to a short duration of estrus and a shorter interval between onset of estrus and ovulation, regardless of parity [59, 60], which increases the risk of suboptimal timing of insemination and can cause the sows to have low farrowing rates and few PBA. Also, especially for parity 1 sows, weaning-to-first-mating interval tends to be increased by low feed intake during lactation and short lactation length [61, 62]. Additionally, there has been progress in genetic programs selecting for decreased weaning-to-first-mating interval which has helped to reduce the probability of a return [63].

#### Abortion in parities 0 and 1

Abortion is maternal failure in some sows [64] due to endocrine disturbances or uterine problems or infection agents such as porcine parvovirus and porcine reproductive and respiratory syndrome virus [56]. It has been reported that 43–44% aborted sows in parities 0 and 1 were culled without any re-service [24], presumably because it was thought that aborted sows were more likely than non-aborted sows to abort in a later pregnancy, and so they have lower longevity than non-aborted sows. However, no large differences were found between the aborted sows and non-aborted sows for subsequent farrowing rate, weaning-to-first-mating interval or lifetime performance [24]. Also, only 4.1% of sows re-serviced after a first abortion experienced a second abortion in the same or a later parity. So, it is recommended that producers re-service aborted sows at the second return to estrus, just like non-aborted sows.

#### Peri-farrowing and lactation care in parity 1

High summer temperature during farrowing increased sow mortality in low parity [65]. A cooling pad has been developed that efficiently removes excess of heat from lactating sows [66]. So, advanced equipment in farrowing barns and increased care during the peri-partum and lactation period could help decrease sow mortality and increase longevity.

Also, decreased feed intake during lactation in parity 1 sows has been clearly associated with lower adjusted 21-day litter weights, prolonged weaning-to-first-mating interval, lower farrowing rate and fewer PBA at subsequent parity and an increased risk of culling due to anestrus or reproductive failure [19, 67–69]. So, it seems clear that increasing feed intake during lactation in parity 1 sows increases longevity, fertility, prolificacy and efficiency of sows.

#### Lameness occurrences

Sows culled due to lameness have lower longevity, lower fertility, lower prolificacy and lower efficiency than those culled for other reasons [5, 12, 70]. Lameness increased the risk of culling from a herd [71], and it was also found that sows with leg lesions were likely to farrow more mummified fetuses and stillborn piglets than those without lesions [70].

Approximately 70% of the sows culled due to lameness were farrowed sows, whereas only 30% were serviced sows. Culling due to lameness frequently occurred 4–8 weeks after farrowing and 4–5 weeks after service, whereas risk factors were high parity and being re-serviced [72]. Therefore, producers are recommended to identify sows with early signs of lameness and move them to a sick pen for recovery [73]. Also, management options that have been shown to protect against lameness include non-slated floors, dry and clean floors and the use of bedding for group housed gilts [71, 73, 74]. Furthermore, management during gilt development should provide appropriate nutrients and energy needed for required growth, as well as bones and reproductive tract development [75].

#### Birth cohort effects

Birth year or herd-entry year effects sow performance and longevity. Genetic progress has enabled sow reproductive performance to improve in recent birth years or herd-entry years. For example, over the last few decades, PBA per sow has been increasing in breeding herds [6, 20, 76].

Birth year or herd entry year has also affected culling due to lameness in herd-entry cohorts, with the incidence rate for culling increasing by 25% from 2011 to 2013 [72]. One possible reason for the increased culling of lame sows that were entered into herds from 2013 is that pregnant sows in group housing tend to exhibit more lameness than those in individual stalls [77–79], and group housing for gestating sows in mid-late pregnancy has been mandatory in the E.U. since January 2013 [80].

### Herd-level factors for lifetime performance

Herd-level factors, including herd size, herd performance and management practices, are associated with lifetime performance of sows. Data relating to management practices can be collected by survey questionnaires, because producers do not commonly record such herd-level information in their recording software. Also, group level or herd-level factors have been shown to interact with sow level predictors for sow lifetime performance [11, 81, 82].

#### Herd size

A study of Spanish herds showed that the number of PBA in parity 1 increased by 0.3 pigs, as herd size increased from 180 to 1300 sows [11]. The reason for this increase could be that large herds have more rapid genetic improvement, better health status or better production systems with advanced facilities than small herds [83, 84]. So, herd size may be an indicator of how advanced a production system is, in terms of the amount of investment, the quality of the facilities, human resources and the level of genetic improvement, although the study of the Spanish herds did also show that large herds had lower longevity of sows than small-to-mid herds [11].

**Fig. 4:**
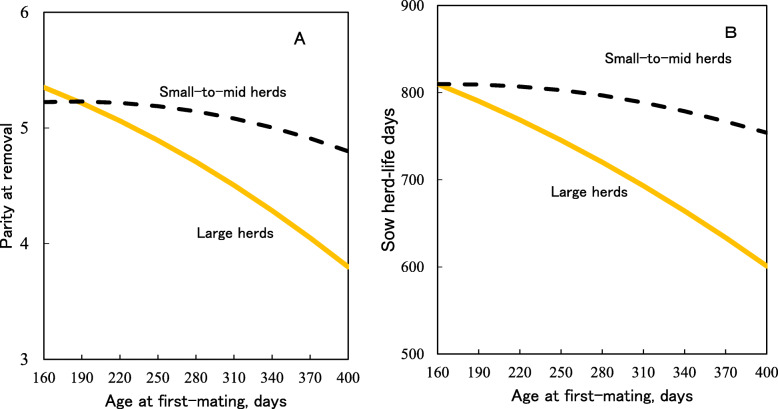
Predicted parity at removal (**a**) and sow herd-life days (**b**) at different gilt ages at first-mating [11]. Herds were categorized into two groups based on the 75th percentile of farm means of herd size: large (≥1,017 sows) or small-to-mid herds (<1,017 sows)

Late AFM interacting with herd size has been shown to decrease sow longevity, prolificacy, fertility and efficiency of sows more in large herds than in small-to-mid herds [11]. Figure 4 shows that increased AFM decreased sow longevity 3–4 times more in sows in large herds than those in small-to-mid herds. It appears that the herd size alters the impact of AFM on sow longevity and lifetime performance.

#### High-performing herds

The concept of high-performing herds based on PSY has been used to provide target values for reproductive performance and productivity in breeding herds [83, 84]. The high productivity of high-performing herds is attributable to better replacement gilt development [27, 84, 85], better breeding management [84, 85], more advanced technologies such as cooling systems, post cervical AI or assisted colostrum intake [86, 87] and better piglet care during lactation [87, 88]. For example, as outside temperature increased from 25 to 35 C^o^, the weaning-to-first-mating interval of sows in high-performing herds increased by only 0.3 days, compared with an increase of 0.8 days in ordinary herds [81]. So, approximately 60% (0.5 days /0.8 days) of the negative effects of high temperature on weaning-to-first-mating interval was able to be alleviated by the management in the high-performing herds.

**Fig. 5:**
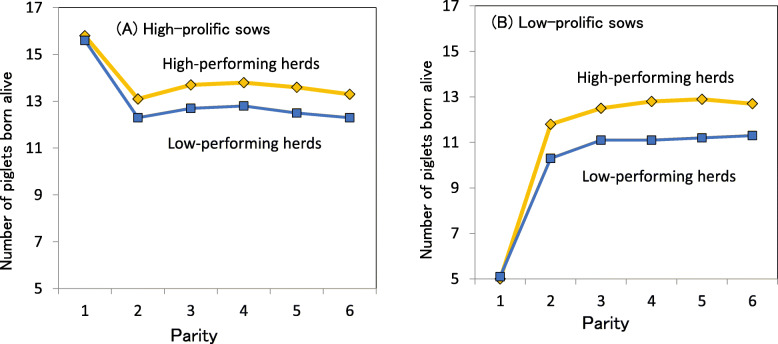
Comparisons between the herd group of the number of piglets born alive of either high-prolific (**a**) or low-prolific sows (**b**) across parities [82]. The two farm groups were categorized by the 25th and 75th percentiles of herd means of herd-life annualized PW, whereas two sow groups were grouped by the 25th and 75th percentiles of the numbers of piglets born alive parity 1

High-performing herd effects interacting with PBA in parity 1 alter both the reproductive potential of sows across parities and their lifetime performance. Figure 5 shows comparisons of PBA by high-prolific and low-prolific sows in either high- or low-performing herds [82]. In parities 2–6, the high- and low-prolific sows in the high-performing herds had respectively 0.8–1.1 and 1.4–1.7 more PBA than the equivalent sow groups in the low-performing herds.

#### Timing of insemination in parity 0

Analysis of survey data has shown that gilts in herds that perform first insemination immediately after first detection of estrus have a higher farrowing rate than those in herds that perform later insemination [18, 89]. A possible reason for the difference in farrowing rates is that gilts with delayed insemination timing might have had a shorter estrus duration, and so would be more likely to have suboptimal timing of insemination [40]. Consequently, the lower farrowing performance of such gilts with delayed insemination would result in suboptimal lifetime performance, such as low longevity, due to early culling for low fertility. Therefore, if a low farrowing rate problem is diagnosed as being related to insemination timing, it is advisable for farm staff to try for an earlier timing of insemination.

#### Culling guidelines

Increased culling interval increases sow nonproductive days [4, 5], decreases fertility and decreases efficiency. In a herd survey, the mean culling guidelines for unmated gilts and for mated gilts due to conception failure were 300 days of age and 37 days from the last mating date, respectively [90]. Furthermore, actual culling intervals for mated gilts were at least 30 days longer than the guideline for culling interval. So, optimizing culling intervals using appropriate herd culling guidelines could improve fertility and efficiency of the sows within the herd.

### Farm data analysis as an observational study

Farm data analysis is an epidemiological observational study. As an observational study, there are no true replicates which controlled experiments should have. However, an observational study with appropriate statistical models can provide practical and readily applicable information to swine veterinarians and producers about real world events and practices that are difficult to investigate using controlled experiments. Also, an observational study is a powerful tool to quantify associations between outcomes and possible risk factors using big data from digital technologies [1].

There are sow level and herd-level farm data analyses. At sow level, sow performances differ in genetics, weaning weight, raising environment, parity, season, farm staff and management practices. So, observational studies of actual farm situations are useful to cope with the variation in sows on farms with different conditions. Also, sow data within a herd could fit to a two-level mixed model, because sows are not independent of the herd having different situations. Additionally, a longitudinal study of sows in farm data analysis should treat parity records in a 3-level structure: parity records within the sow in the herd.

Herd-level analysis is simple and can be useful for exploring a time trend or for providing an overview of herd performance over a long period, because there could be large changes in the management, health conditions and genetics of the herd over the study period, for example over a 10-year period. However, with herd-level analyses, it is not possible to perform multivariate analyses to account for the variation due to sow level factors such as parity, season, lactation length, herd-entry year, and their interactions; whereas these analyses can be performed with sow level analysis.

## Discussion

We have defined and organized the four components in sow lifetime performance in two tree forms. In the sow lifetime performance trees, annualized PW and annualized PBA measured as lifetime efficiency are proposed as integrated measurements for sow lifetime performance.

Probably, annualized PBA is better than annualized PW for measuring a sow’s lifetime performance without taking management or facility impacts into account, especially when considering the use of nurse sows, fostering practices or advanced facilities (e.g. milk replacer feeders) in commercial herds. However, annualized PBA does not cover lactational performance details, including milking capability. Also, while adjusted 21-day litter weight is a measure of milking capability in farm data analysis [4, 5], it is not commonly recorded.

For longevity, sow life days can be an appropriate measurement for producers who raise replacement gilts from birth to removal on the same farm or within the same production system. In such systems, the number of days taken to raise the gilts should be counted toward longevity. In contrast, herd-life days is a useful measurement for producers who purchase replacement gilts from outside breeding companies where the cost is the price per gilt, rather than the price per day of gilt age. However, it should be noted that sow longevity may not be critical for large producers who purchase a replacement gilt batch based on PSY which the batch will produce.

Among many predictors for high lifetime performance in this review, age at first-mating is commonly measured and might be the most useful for predicting sow lifetime performance. Also, there is a link between early sexual maturity in gilts and aspects of high lifetime performance of sows such as high prolificacy, fertility and longevity [3, 27, 33]. Consequently, producers are recommended to implement effective gilt development programs with a boar exposure area in order to stimulate pubertal estrus and increase the number of early maturing gilts [27, 33].

PBA in parity 1 is a simple and powerful predictor for high prolificacy including more PBA in later parities. However, PBA in parity 1 should be carefully used as a predictor of sow lifetime performance, because some sows with large litter size have litters with extremely light birth weights which are associated with low survival and slow growth performance post weaning. A breeding herd is a provider of piglets weaned for nursery, grower and finisher phases [4]. Thus, breeding herds should produce sows that will sufficient numbers of PBA and that will produce piglets of high quality or high potential growth performance.

It should be noted that no difference was found in weaning-to-first-mating interval or adjusted 21-day litter weight between prolificacy groups based on PBA in parity 1 [27, 42], which suggests that less prolific sows are not inferior in terms of fertility or milk production. Prolificacy appears to be independent of either fertility or milking capability. So, low prolificacy does not mean either low fertile or low lifetime performance. Such low prolific but high fertility sows can take foster piglets from sows that have farrowed surplus piglets. Also, sows with high prolificacy or high fertility are more likely to have high longevity and high efficiency [27, 42, 58].

Finally, sow data, sensor data and behavior data that have recently started being collected by digital technologies will provide a large opportunity for producers and veterinarians to monitor and care for individual sows in breeding herds [1, 80]. Such big data collected on farms should be transformed into valuable information by farm data analysis to improve the making decision process for maximizing sows’ potential, optimizing their lifetime performance and enhancing animal welfare in breeding herds. Farm data analysis can also be utilized to examine risk factors for pre-weaning mortality and the number of stillborn piglets. These are two of the most urgent areas that need further research because our tree for annualized PW indicates that the average sow loses 25 piglets or more during its lifetime as stillborn or pre-weaning dead piglets.

## Data Availability

Not applicable.

## Abbreviations

AFM: Age of gilts at first-mating
PBA: Piglets born alive
PSY: The number of piglets weaned per sow per year
PW: Piglets weaned
